# Rotavirus genome replication: Some assembly required

**DOI:** 10.1371/journal.ppat.1006242

**Published:** 2017-04-20

**Authors:** Courtney P. Long, Sarah M. McDonald

**Affiliations:** 1 Virginia Tech Carilion School of Medicine and Research Institute, Roanoke, Virginia, United States of America; 2 Translational Biology, Medicine, and Health Graduate Program, Virginia-Maryland College of Veterinary Medicine, Blacksburg, Virginia, United States of America; 3 Department of Biomedical Sciences and Pathobiology, Virginia-Maryland College of Veterinary Medicine, Blacksburg, Virginia, United States of America; University of Michigan Medical School, UNITED STATES

## Introduction

Viruses are obligate intracellular parasites comprised of a nucleic acid genome (RNA or DNA) that is encased within a proteinaceous capsid particle and/or lipid envelope. Genome replication and virion assembly are central processes in the life cycles of all viruses. In many cases, a virus will first make multiple copies of its genome and then subsequently package those copies into newly formed viral capsids or envelopes. However, rotaviruses and other members of the Reoviridae family differ in that they replicate their genomes in concert with virion assembly. Specifically, the segmented, double-stranded RNA (dsRNA) rotavirus genome is copied within a subviral assembly intermediate that goes on to become a mature, infectious virion. A key feature of this replicase–assembly process is that the rotavirus polymerase (VP1) is only active when tethered to the core shell protein (VP2) within the confines of an assembly intermediate. Yet several gaps in knowledge exist about the structure and composition of early assembly intermediates for rotavirus, and the mechanism by which VP2 engages and activates VP1 is not completely understood.

## What are the structures and functions of rotavirus triple- and double-layered particles?

**Fig 1:**
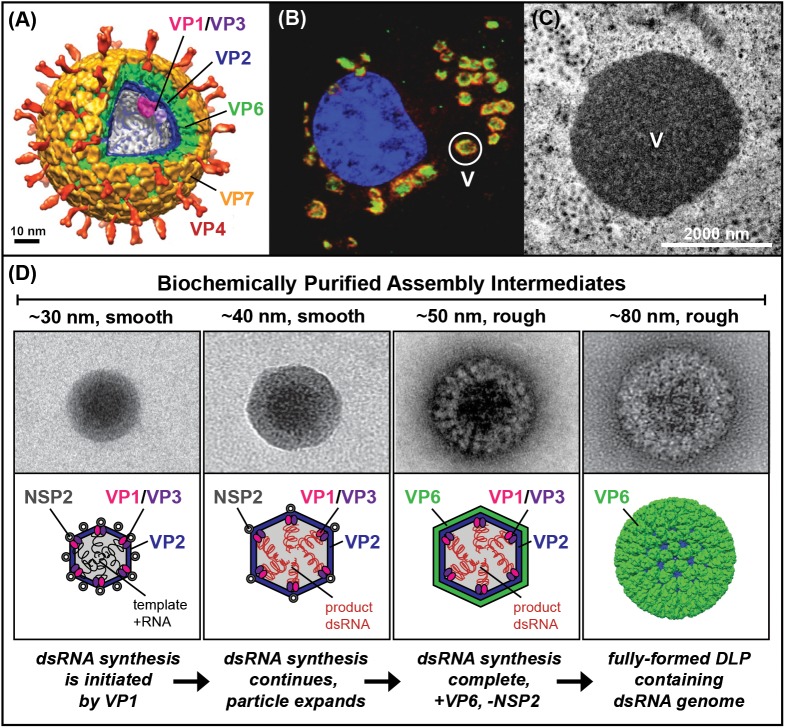
Rotavirus virion, viroplasms, and one hypothetical model of the replicase–assembly pathway. (A) Structure of the triple-layered rotavirus virion [1]. Locations of viral proteins are indicated. VP1 and VP3 are modeled into the structure. (B) Confocal micrograph of a rotavirus-infected monkey kidney cell. The cell was stained to show the locations of VP2 (red), NSP2 (green), and the nucleus (blue). A single viroplasm (V) is outlined with a white circle. (C). Electron micrograph of resin-embedded, negatively-stained cell section. A single, electron-dense viroplasm (V) is shown in the center. (D) Electron micrographs of biochemically purified assembly intermediates and one hypothetical model of early rotavirus assembly. Beneath each micrograph is a cartoon representation (or, in the case of the DLP, a structure) that depicts the possible composition of the assembly intermediate, and the images are ordered according to one hypothetical pathway of early virion morphogenesis. The protein composition and activity of each particle type has not been experimentally validated.

The rotavirus virion is a triple-layered particle (TLP) ~85 nm in diameter, and it is made up of a VP2 core shell, a middle VP6 layer, and an outer VP7 layer that is embedded with VP4 spike attachment proteins (**Fig 1A**) [1–2]. It is thought that the VP1 polymerase forms a complex with the VP3 RNA capping enzyme and that VP1–VP3 heterodimers are tethered to VP2 beneath each of the 12 icosahedral fivefold axes [3]. The rotavirus genome is made up of 11 dsRNA segments, coding for 6 structural proteins (VP1–VP4, VP6, and VP7) and 6 nonstructural proteins (NSP1–NSP6) [4]. The TLP is the infectious form of the virus that attaches to and enters into host cells. However, during the cell entry process, the outer VP4–VP7 layer of the TLP is shed, depositing a double-layered particle (DLP) into the cell cytoplasm. VP1 polymerases within the DLP synthesize single-stranded, positive-sense RNAs (+RNAs), which acquire a 5’ cap structure (m^7^GpppG) by the activities of VP3 [5]. These +RNAs serve as mRNA templates for protein synthesis, and they are also selectively assorted and packaged into an early assembly intermediate where they serve as templates for genome replication by VP1 (see details below) [6]. The mechanism by which rotavirus acquires one of each of its 11 genome segments is poorly understood, yet studies of other Reoviridae members suggest that this assortment process is mediated by RNA–RNA interactions among the single-stranded transcripts [6–7].

## What are the structures and functions of early rotavirus assembly intermediates?

Atomic resolution structures have been determined for rotavirus TLPs and DLPs, revealing exquisite details about capsid protein organization [1–2, 8]. By contrast, much less is known about the structures of early, replicase-competent assembly intermediates for rotavirus. One reason for our lack of knowledge about these particles is the fact that they are encased within viroplasms, which are discrete, cytoplasmic inclusions ~1–3 μm in diameter (**Fig 1B**) [9]. At <100 nm in diameter, assembly intermediates are too small to be seen using conventional light microscopy, and unfortunately, viroplasms are so electron-dense that internal features can’t be resolved by higher-resolution electron microscopic (EM) imaging (**Fig 1C**). Subviral particles capable of mediating in vitro dsRNA synthesis can be isolated from rotavirus-infected cells using biochemical approaches [10–13]. These putative early assembly intermediates contain VP1, VP2, VP3, and VP6, as well as NSP2, a multifunctional viral nonstructural protein critical for viroplasm formation and genome replication [14]. When viewed using negative-stain EM, the isolated particles are heterogeneous in their sizes and features (**Fig 1D**) [13]. Specifically, the smaller particles (~30–40 nm in diameter) exhibit smooth borders, whereas the larger particles (~50–70 nm in diameter) show a rough, honeycomb pattern on their surface, reminiscent of 80-nm DLPs. Unlike DLPs, however, the isolated assembly intermediates are very fragile and highly permeable to metal stains and RNases, suggesting that they do not have fully formed capsid layers. One hypothetical model of the rotavirus replicase–assembly pathway is that the smaller, smooth particles turn into the larger, rough particles and ultimately into DLPs (**Fig 1D**). For instance, the ~30-nm smooth particle could represent the earliest rotavirus assembly intermediate, within which genome replication is initiated by VP2-bound VP1. Biochemical data suggest that each VP1 polymerase functions independently within an assembly intermediate but that 11 polymerases act in synchrony with each other so that the genome segments are synthesized at the same time [11]. It is not known how the activity of one polymerase is coordinated with those of the other polymerases. Nevertheless, as the polymerases convert the +RNAs into dsRNAs, the particle presumably expands and begins to acquire a VP6 layer, forming a larger rough particle. Prior to final DLP assembly, the nonstructural protein NSP2 must be removed. Nascent DLPs egress from the viroplasm and bud into the adjacent endoplasmic reticulum, where they are converted into TLPs by addition of VP4 and VP7 [15–16]. Further studies are required to elucidate higher resolution structures, compositions, and activities of isolated rotavirus assembly intermediates and to test this hypothetical model of early morphogenesis.

## How does the VP2 core shell engage the VP1 polymerase during early particle assembly?

**Fig 2:**
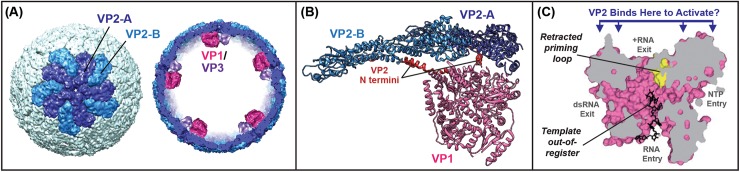
Rotavirus VP1-VP2 interactions. (A) Left: Structure of the rotavirus VP2 core shell in the context of a DLP [5]. VP2-A and VP2-B monomers of one decamer unit are colored blue and cyan, respectively. Right: The core shell is shown computationally sliced through the middle to reveal the likely locations of VP1–VP3 heterodimers. (B) VP1–VP2 contacts in the fully-assembled DLP [17]. VP2-A, VP2-B, and VP1 are shown in ribbon representation and colored blue, cyan, and pink, respectively. The visible regions of the VP2 N termini are colored red. (C) VP1 is shown in surface representation and computationally sliced through the middle to reveal four tunnels [19]. The location of the retracted priming loop is shown in yellow, and a 7-nucleotide +RNA template that is bound out-of-register with the active site is shown in black. VP2 is predicted to engage VP1 at the +RNA exit interface and trigger structural rearrangements including a repositioning of the priming loop and +RNA template.

To initiate genome replication (i.e., dsRNA synthesis), the VP1 polymerase must be bound by the core shell protein VP2. In DLPs or TLPs, VP2 is organized as 12 interconnected, decameric units (**Fig 2A**), but its structural organization in assembly intermediates is not known [1–2,8]. The VP2 monomers in each decamer unit adopt one of two slightly different conformations (VP2-A and VP2-B). One conformation, VP2-A, converges tightly around the icosahedral axis, whereas the other conformation, VP2-B, intercalates between adjacent VP2-A monomers. The extreme N-terminal region of VP2 (residues ~1–100) protrudes inward and makes contact with VP1 (**Fig 2B**). Estrozi et al. predicted that VP1 is positioned against the inner surface of the VP2 core shell off-center from the fivefold axis and that it is stabilized against the core shell by VP2 N termini [17]. The regions of VP2 that contact VP1 in the DLP structure are the same as those shown to be important for VP2-mediated VP1 enzymatic activation in vitro [18]. This observation suggests that the VP1 and VP2 binding interaction during the early replicase–assembly process may be similar to the VP1 and VP2 binding interaction during transcription.

VP1 is a globular, cage-like enzyme comprised of a central polymerase domain (with canonical finger, palm, and thumb subdomains) that is flanked by extended N- and C-terminal domains [19]. Together, these three domains create four distinct tunnels within VP1 that support nucleoside triphosphate (NTP) entry, +RNA entry, +RNA exit, and dsRNA exit (**Fig 2C**). Within the DLP, VP1 is proposed to be oriented with its +RNA exit tunnel facing towards the VP2 core shell; such an orientation would facilitate the egress of +RNAs from the DLP capsid layers during transcription (**Fig 2C**) [5]. This orientation of VP1 is also similar to that proposed for the polymerases of other Reoviridae family members [20–21]. However, the importance of VP2 binding to the VP1 +RNA interface for VP1 replicase function has yet to be biochemically tested. It also remains unclear how VP2 engagement of VP1 leads to enzymatic activation of the polymerase. The structure of VP1 in complex with template +RNA—but in the absence of VP2—reveals an auto-inhibited, inactive polymerase with at least two malpositioned elements: the so-called “priming loop” of VP1 (which stabilizes the initiating NTP during genome replication) is bent too far forward, and the +RNA template is bound out-of-register with the active site (**Fig 2C**) [19]. Thus, VP2 engagement of VP1 may trigger a series of conformational changes in the polymerase interior, including repositioning the priming loop and +RNA template to allow for the initiation of dsRNA synthesis. Further studies seeking to test this hypothesis will benefit from a robust in vitro assay that recapitulates VP2-dependent VP1 activation [22–23].

## Perspectives

During their intracellular life cycles, viruses make numerous copies of their nucleic acid genomes and package them into nascent particles. Viral genome replication and particle assembly are often highly coordinated within the infected cell to maximize efficiency. Rotaviruses and other Reoviridae family members may very well exhibit the utmost level of coordination, as they replicate their genomes concurrent with assembly of new virions. The mechanism of this concerted replicase–assembly process is not completely understood. Isolated rotavirus subviral particles that can perform dsRNA synthesis in vitro are just beginning to be characterized in terms of their structure and composition, and there is much to be learned about how the activity of the rotavirus VP1 polymerase is regulated via interaction(s) with the core shell protein VP2 in the context of the assembling particle. Although other viruses do not perform this same multitasking feature of replicating their genomes while assembling particles, it is apparent that they also must regulate the activities of their polymerases. The vast majority of viral polymerases do not function as sole polypeptides during infection [24]. Instead, they are components of multisubunit complexes, and interactions between the protein constituents dictate the polymerase activity. Thus, studies of rotavirus polymerase regulation during particle assembly may broadly inform an understanding of how other viruses ensure that genome replication occurs at the right place and time in the infected cell.
